# A Review of a Report of a Committee of the American Medical Association, on the Permanent Cure of Reducible Hernia

**Published:** 1853-08

**Authors:** 


*A Review of a Report of a Committee of the American Medical Association, on the Permanent Cure of Reducible Hernia.* By GEORGE HEATON, M. D.

Messrs. Hayward, Warren, and Parkman, the committee alluded to in the title, addressed a note to Dr. Heaton, requesting an account of his operation for the radical cure of hernia, to be embodied in their report. This note was courteous and complimentary. Dr. Heaton declined to communicate his method, alledging want of time, insufficiency of space, and his asserted intention of publishing a work on the subject, as his excuses for non-compliance. The only knowledge we have of the matter, is from the pamphlet before us. The question involved is this. Did Dr. Heaton refuse to communicate his mode of cure? He himself admits that he did. He excuses this mal-feasance, by denying the right of the committee to avail itself of his long and arduous labors. The excuse is insufficient. There is a sacred principle of honor, of common justice to humanity, and common decency to professional brethren, that requires from the highest minds of the profession, that they should freely, and for the love of science and humanity, communicate to the humblest tyro any information asked for. Upon one who, like Dr. Heaton, claims to have discovered a cure of the last importance, the obligation to give it wide publicity rests still more strongly. Dr. Heaton, after speaking of many years devoted to this branch, and mentioning positively his success, still professes a fear of publishing something that may not stand the test of time. Again, we say, his excuse is jejune—most unmistakably an evasion. Life is uncertain. Dr. Heaton may die to-morrow; and his secret with him. His death, under such circumstances, would be constructive larceny, for he would filch with him from the living world, that which is its due—his *life*, under the same circumstances, is —— ——.

We cannot resist the conclusion that, after all this blowing of his own trumpet, Dr. Heaton *has* no discovery—that he has revamped some antiquated mode, which he does not dare to submit to the inquisition of an intelligent and honorable profession. S. B. H.

